# Aerogels are not regulated as nanomaterials, but can be assessed by tiered testing and grouping strategies for nanomaterials†

**DOI:** 10.1039/d1na00044f

**Published:** 2021-05-19

**Authors:** Johannes G. Keller, Martin Wiemann, Sibylle Gröters, Kai Werle, Antje Vennemann, Robert Landsiedel, Wendel Wohlleben

**Affiliations:** BASF SE, Dept. Experimental Toxicology and Ecology, Dept. Material Physics 67056 Ludwigshafen Germany wendel.wohlleben@basf.com; IBE R&D Institute for Lung Health, gGmbH Münster Germany

## Abstract

Aerogels contribute to an increasing number of novel applications due to many unique properties, such as high porosity and low density. They outperform most other insulation materials, and some are also useful as carriers in food or pharma applications. Aerogels are not nanomaterials by the REACH definition but retain properties of nanoscale structures. Here we applied a testing strategy in three tiers. In Tier 1, we examined a panel of 19 aerogels (functionalized chitosan, alginate, pyrolyzed carbon, silicate, cellulose, polyurethane) for their biosolubility, and oxidative potential. Biosolubility was very limited except for some alginate and silicate aerogels. Oxidative potential, as by the ferric reduction ability of human serum (FRAS), was very low except for one chitosan and pyrolyzed carbon, both of which were <10% of the positive control Mn_2_O_3_. Five aerogels were further subjected to the Tier 2 alveolar macrophage assay, which revealed no *in vitro* cytotoxicity, except for silicate and polyurethane that induced increases in tumor necrosis factor α. Insufficiently similar aerogels were excluded from a candidate group, and a worst case identified. In the Tier 3 *in vivo* instillation, polyurethane (0.3 to 2.4 mg) elicited dose-dependent but reversible enzyme changes in lung lavage fluid on day 3, but no significant inflammatory effects. Overall, the results show a very low inherent toxicity of aerogels and support a categorization based on similarities in Tier 1 and Tier 2. This exemplifies how nanosafety concepts and methods developed on particles can be applied to specific concerns on advanced materials that contain or release nanostructures.

## Abstract 2

Aerogels may interact on multiple scales. Here we develop a testing strategy and potential categorization of 17 organic and 2 inorganic aerogels based on biosolubility, oxidative potential, *in vitro* reactivity and *in vivo* instillation.

## Graphical abstract

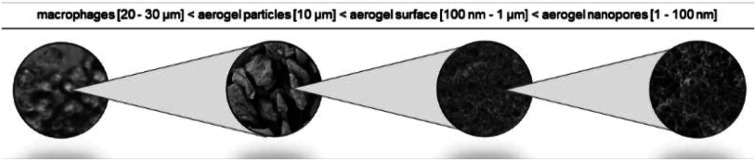

## Introduction

Aerogels are unusual materials that combine macroscopic external and nanosized internal structures with high specific surface area, low density and high porosity. Aerogels are often used as advanced insulation materials,^1,2^ or as innovative tools for the microencapsulation of additives and drugs in food and pharmaceuticals.^3–6^ Precipitated or fumed silica aerogels, which are very hygroscopic, are also used as desiccants^7,8^ or as insecticide against drywood termites or flour beetles.^9,10^ Aerogels and other novel foams are promoted as “advanced materials”.^11–14^ However, the commercialization of aerogels in products may lead to an exposure of consumers to fragments of aerogels, either *via* pulmonary or oral uptake. The risk for humans may be low, as long as the hazard potential of inhaled or swallowed aerogel beads or fragments is low as well. However, the development of appropriate methods to characterize exposure and hazard of aerogel beads is challenging because they retain properties of the nanoscale only by their internal structure. Hence, aerogels might be in the scope of the labelling provisions for nanomaterials provided by the EU Novel Foods Directive. On the other hand, internally structured porous materials such as aerogels are explicitly excluded^15,16^ from the need to register a “nanoform” under the revised REACH Annexes. Aerogels are also exempted from the need to report to national nanomaterials product inventories such as those in France, Belgium, the USA or Canada.^17,18^ Nevertheless, producers cannot neglect that, although the inherent toxicity of most materials that can be formulated as an aerogel is low, an increased bioactivity may result from their large inner surface area, possibly fostering a high surface reactivity and/or a high dissolution.^19^ Toxicity inherent to nanostructures is not expected in general,^20^ but the hazard assessment of aerogels needs to consider the composition of inhalable or ingestible fragments, and the modulation of their potency by the large surface area.

In any case the testing strategy should be derived from the intended use. While the oral uptake pathway plays a minor role for most materials, unintended inhalation of aerogel dusts followed by pulmonary deposition is considered the most critical route of exposure.^21^ Especially the installation and removal of insulation materials in houses almost inevitably entails an occupational exposure to inhalable aerogel fragments. This application is an industrial reality already for silica- and PU-based aerogels. Powder handling is required as well during the manufacturing of consumer goods of alginate, chitosan and other aerogels, and may induce other occupational exposures. Only for silica desiccant gels, which are related to aerogels and may contain respirable fragments, limited data on toxicological effects is available from the early beginnings of the aerogel development.^22–24^ Inhalation experiments have shown that silica desiccant gels induce adverse effects only at a relatively high concentration (>1 mg m^−3^) and long-term feeding studies found no evidence for tumor induction.^25,26^ However, the hazard potential of most aerogels is unknown,^27^ except for seminal studies that focused on their biomedical uses,^28^ specifically as scaffold implants^29,30^ and other studies that focused on the bioavailability of drugs or biocides carried by aerogels.^31–33^

**Fig. 1:**
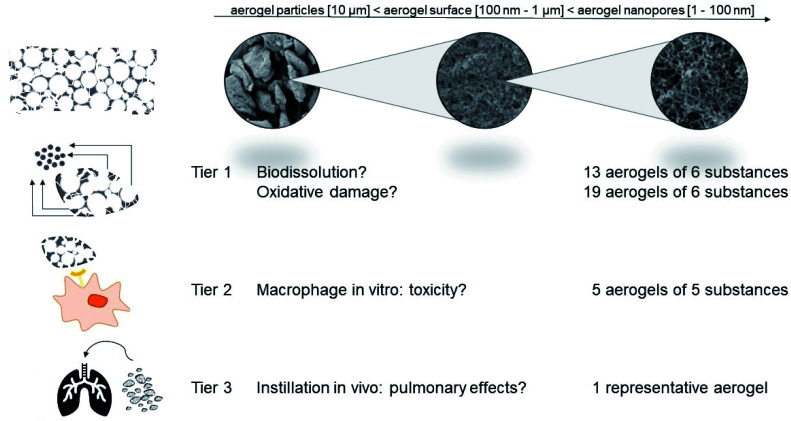
Test strategy: Tier 1 and Tier 2 apply biophysical and *in vitro* toxicity screenings to identify a potential category of aerogels that are sufficiently similar in their biological interactions. Materials with a distinct behavior are removed from the category and may require individual assessment. Tier 3 assesses the *in vivo* pulmonary effects of an aerogel that is representative of the potential category, by instillation into the rat lung.

Here we explore a tiered testing strategy derived from a categorization and grouping perspective specifically for nanomaterials.^34–36^ Regulators explicitly support such approaches to avoid unnecessary animal testing.^37,38^ The key idea is that safety is demonstrated by establishing a hypothesis of similarity (why should a group of different materials behave similarly?), to assess the candidate group by targeted testing, and then to remove materials that are not sufficiently similar (Fig. 1).^39^ Importantly, we include benchmark materials that represent more conventional porous materials, specifically pyrolysed carbon (related to activated charcoal) and silica aerogels on Tier 1 (abiotic screening, Fig. 1). For organic aerogels, tests on Tier 2 (*in vitro* toxicity) enhance the reliability of the assessment, which is finally calibrated for a representative aerogel by Tier 3 (*in vivo* toxicity).

## Results and discussion

### Testing strategy

The testing strategy complies with the recent GRACIOUS framework for the grouping of nanomaterials,^40^ and the specific tests comply with the tiered selection of methods in GRACIOUS inhalation grouping, with the nanoGRAVUR grouping framework,^41^ and with the tiered DF4nanoGrouping,^42^ which were tested in case studies including silica and organic nanomaterials.^41,43^ All of these tiered frameworks were previously applied to materials with *external* dimensions in the nanometer range only, whereas here we apply them to open-pore *internal* nanostructures. The large surface area is the common feature of both classes of nanomaterials.

### Abiotic reactivity

**Fig. 2:**
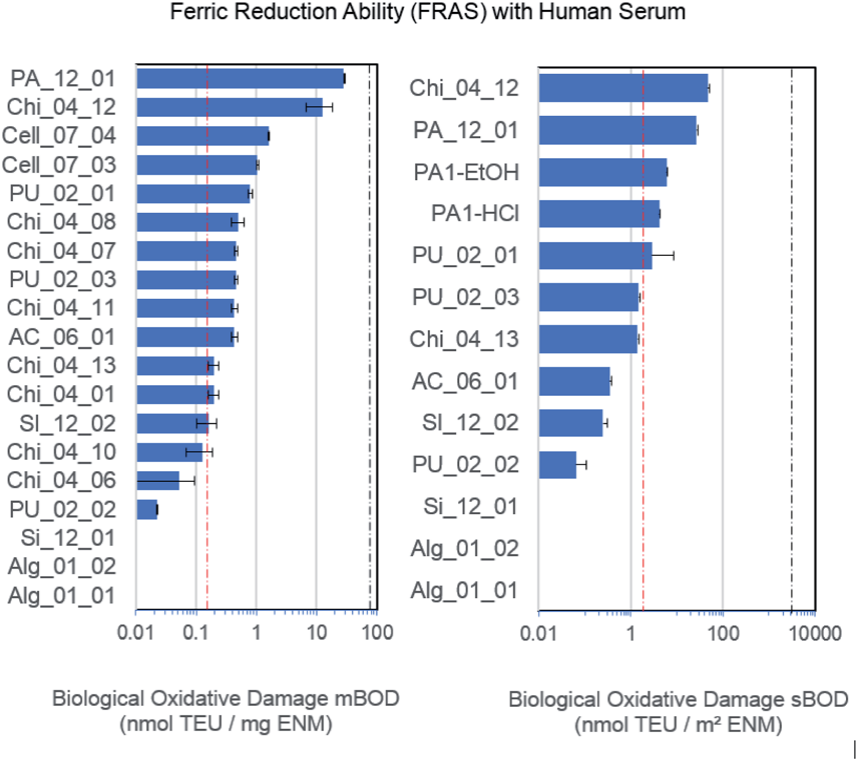
Surface reactivity measured with the FRAS assay. Data are presented as mass based biological oxidative damage (mBOD, left). For a subset of the data the surface-based biological oxidative damage was calculated (sBOD, right); an average specific surface area was used for the negative control. No displayed bar (Si_12_01, Alg_01_02 and Alg_01_01) corresponds to no significant signal. The red and black dashed lines display the negative and positive control respectively.

The FRAS assay detects damage to antioxidants in human serum.^44–46^ Oxidative damage can be expressed as mass-based biological oxidative damage (mBOD), or as a surface-based biological damage (sBOD), normalised by the specific surface area (s. Table 1 for BET values). However, as the inner surface may not be fully accessible to bio-molecules, mBOD appears as the more conservative metric. One of the relevant antioxidants in human serum, alpha-tocopherol molecule (vitamin E), fits into these pores, but the diffusion time to permeate the porous network may exceed the 3 h incubation time. In comparison to the positive (Mn_2_O_3_) and negative control (particle free), only the functionalized chitosan Chi_04_12 and the pyrolyzed carbon PA_12_01 showed a significant mBOD (Fig. 2). However, in the sBOD metric five materials exceeded the negative control, and again functionalized chitosan Chi_04_12 and pyrolyzed carbon PA_12_01 gave significant results, whereas hemicellulose materials PA1-EtOH and PA1-HCl and also one polyurethane (PU_02_01) were slightly elevated. Of note, the different production pathways of polyurethanes apparently led to significant differences in reactivity. Thus, the sBOD ranged from 0.06 nmol TEU per m^2^ for PU_02_02 up to 2.83 nmol TEU per m^2^ for PU_02_01 (Fig. 2). All tested polyurethane aerogels share the same chemical composition but differ with respect to production process especially in porosity and grain size. The porosity may influence the reactivity, since it determines the biologically accessible surface area.

**Table 1:** Composition and specific surface area of 19 test materials

Sample	Synthesis	Composition	BET [m^2^ g^−1^]
Chi_04_10	DLR	Functionalized chitosan aerogel	80
Chi_04_11	DLR	Functionalized chitosan aerogel	28
Chi_04_01	DLR	Functionalized chitosan aerogel	169
Chi_04_13	DLR	Functionalized chitosan aerogel	310
Chi_04_12	DLR	Functionalized chitosan aerogel	273
Chi_04_06	DLR	Chitosan control type 1	6
Chi_04_07	DLR	Chitosan aerogel type 2	29
Chi_04_08	DLR	Functionalized chitosan aerogel	37
Alg_01_01	TUHH	Alginate aerogel	613
Alg_01_02	TUHH	Alginate aerogel	560
AC_06_01	Dräger	Activated carbon from coconut shell	1300
PA_12_01	NKUA	Pyrolyzed carbon	1060
Cell_07_03	ARMINES	Pulp aerogel low hemicellulose concentration – ethanol coagulation	285
Cell_07_04	ARMINES	Pulp aerogel low hemicellulose concentration – HCl coagulation	388
PU_02_03	BASF	Polyurethan foam Taber	335
PU_02_01	BASF	Polyurethan foam Taber	369
PU_02_02	BASF	Polyurethan foam milled	350
Si_12_01	NKUA	Subcritical silica	959
SI_12_02	NKUA	Subcritical silica	805

In other cases, different aerogels of closely related chemical composition were found to have a very similar reactivity, such as the two different hemicellulose materials, PA1-HCl as well as PA1-EtOH despite their difference in surface area of 48 m^2^ g^−1^. Similarly, the different chitosan compounds Chi_04_07 and Chi_04_08 as well as Chi_04_13, Chi_04_10, Chi_04_11, Chi_04_01 exhibited a mBOD within the same order of magnitude. Chi_04_06 was used as the reference sample (not an aerogel) and consisted of pure chitosan powder with 90% deacetylation. As expected, it has the lowest reactivity of all forms of chitosan (Fig. 2). Interestingly, even after rescaling the reactivity from mass dosimetry to surface dosimetry, the significant difference between Chi_04_12 and Chi_04_13 remained (Fig. 2). Both were prepared by the same procedure, only replacing hydrochloric acid by acetic acid in the preparation of the chitosan solution and gelation. Since the wet gel body was washed with NaOH solution, then with water, then with ethanol, and finally dried in scCO_2_, the observed differences cannot be attributed to the accessible specific surface area or to impurities from the process but must be attributed to actual differences of reactivity of the Chi_04_12 aerogel.

Alginate materials apparently interfered with the assay as they underscored the negative control. This artifact was attributed to the (partial) solubility and adsorption of organics from the human serum.

ECHA guidance suggests grouping of similar nanoforms within the same substance only.^37^ However, the reactivity results suggest that all aerogels with the chemical compositions of cellulose, alginate, silicate and polyurethane (excluding chitosan and pyrolised carbon) range within the negative control and can be grouped by low surface reactivity, for which the aerogel PU_02_01 is a suitable representative material.

### Biodissolution

**Fig. 3:**
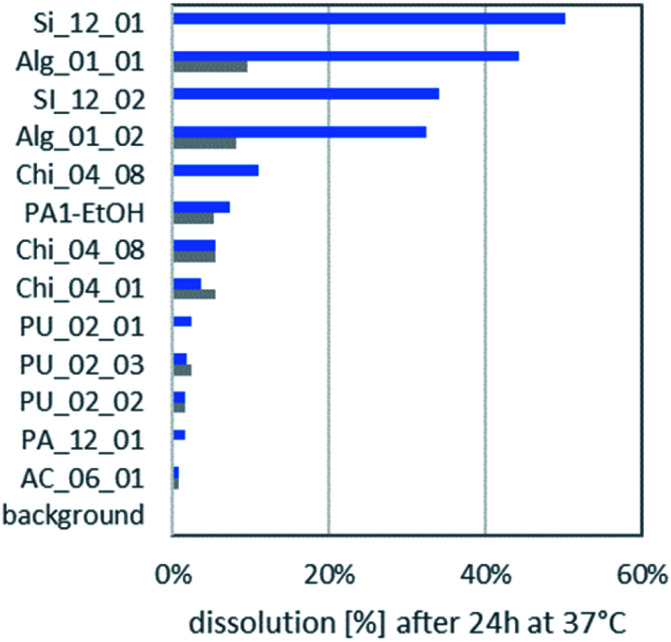
Dissolution of aerogels in pH 4.5 PSF (blue) and pH 1.6 GIF (gray). PSF is relevant for inhalation and mimics the uptake in phagolysosomes *e.g.* by macrophages, which digest foreign material in their lysosome; GIF is relevant for oral uptake and mimics the pH condition of the stomach conditions. Not all aerogels were measured under all conditions.

The dissolution of the aerogels in lysosomal PSF (pH 4.5) and gastric simulant GIF (pH 1.6) is displayed in Fig. 3. In almost all cases aerogels had a higher dissolution in PSF than in the even more acidic GIF. It was noted earlier that release of ketoprofen from alginate and pectin aerogel particles was sensitive to pH.^4^ All aerogels remain far below the EFSA cutoff of 88% dissolution after 10 minutes, which would exempt them from nano-specific assessment.^47^ Even after 24 h incubation, the maximally dissolved fraction reaches only 50%, and the maximum dissolved concentration of the organic aerogels was 27 mg L^−1^ for Alg_01_01.

The most important factor that determined the ranking of biodissolution in Fig. 3 was the molecular composition: all PU aerogels rank low; all silica or alginate aerogels rank high. As secondary factor, both the grain size (related to the outer surface) and the interior structure (which dominates the total specific surface) may modulate dissolution. *A priori* one does not know if the entire interior surface is accessible. However, the relative ranking between the two silica aerogels, and also the relative ranking between the two alginate aerogels correlate to their rankings in specific surface area (Table 1), indicating that the interior surface is decisive for dissolution.

**Fig. 4:**
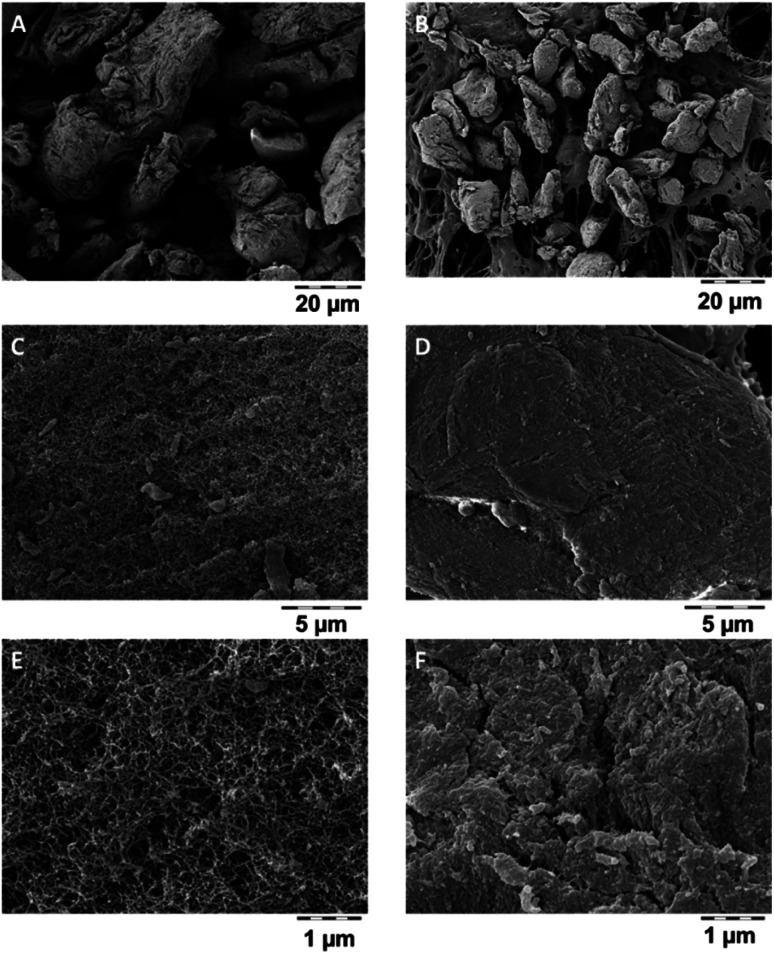
Transformation of PU_02_02 in GIF fluid as detected by SEM. (A), (C) and (E) are images of the pristine material before dissolution studies, (B), (D) and (F) corresponding images after 24 h in GIF.

**Fig. 5:**
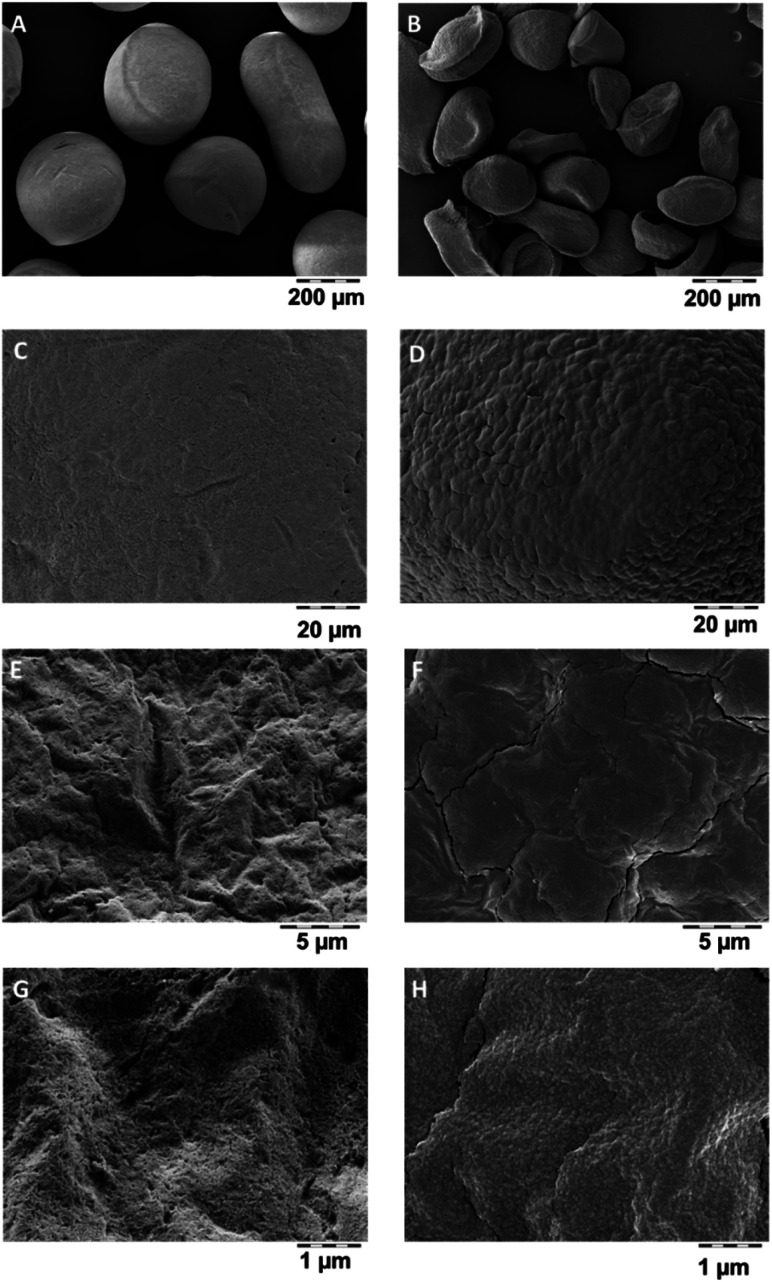
Transformation of Alg_01_01 in GIF fluid as detected by SEM. (A), (C), (E) and (G) are images of the pristine material before dissolution studies, (B), (D), (F) and (H) corresponding images after 24 h in GIF.

Dissolved organics, specifically alginate, pectin, chitosan, cellulose, will become bioavailable, but do not pose a hazard. But also the remaining solids may have lost their internal nanostructures by transformation, even if very little was dissolved. Transformation was tested on a polyurethane-based aerogel, PU_02_02, which had below 5% dissolution (Fig. 3), and which was analysed by SEM before and after incubation in gastric fluid (Fig. 4). The coverage of the aerogel outer surface by components of the GIF medium conceals most of the structure, but cracks (Fig. 4F) allow a peak into the interior, where the nanostructure underneath seemed to remain intact. In contrast, a significantly dissolving aerogel, alginate, showed a an apparent collapse of the external shape, potentially indicative of a collapse of the interior nanostructure (Fig. 5B), and also in magnification showed no indications of remaining porosity (Fig. 5D, F and H). The pores and holes of the pristine material (Fig. 5E and G) are lost after incubation (Fig. 5F and H). The experimental evidence comes with the caveat that observed changes of the structure may be partially induced by the drying *after* incubation, not by the interaction with the physiological medium *during* incubation. We did not succeed to invent a procedure for a physiologically relevant incubation followed by non-invasive solvent exchanges and again supercritical drying. Ideally also one would measure the loss of internal surface area, but nitrogen adsorption would not be reliable due to the unavoidable mixture with components of the simulant media, despite washing. From a hazard screening perspective, collapsing aerogel has lost nanospecific properties, and does not require nano-specific assessment. However, partial stability of an aerogel carrier is required for application as carrier, as the stability improves the release kinetics of low solubility drugs.^6^

### Tier 2: *in vitro* testing of aerogels

The NR8383 alveolar macrophage assay, a Tier 2 testing method (Fig. 1), has been repeatedly shown to provide reliable *in vitro* information on the effects of respirable particles in the lung.^48^ Based on the results of the surface reactivity and dissolution studies, five aerogels (Chi_04_01, Si_12_01, Alg_01_01, TU-HH, AC_06_01, PU_02_02) were chosen for *in vitro* testing with the NR8383 alveolar macrophage assay. In addition quartz DQ12 and corundum particles were used as control particles from well characterized batches.^49^

#### Dosing considerations

**Fig. 6:**
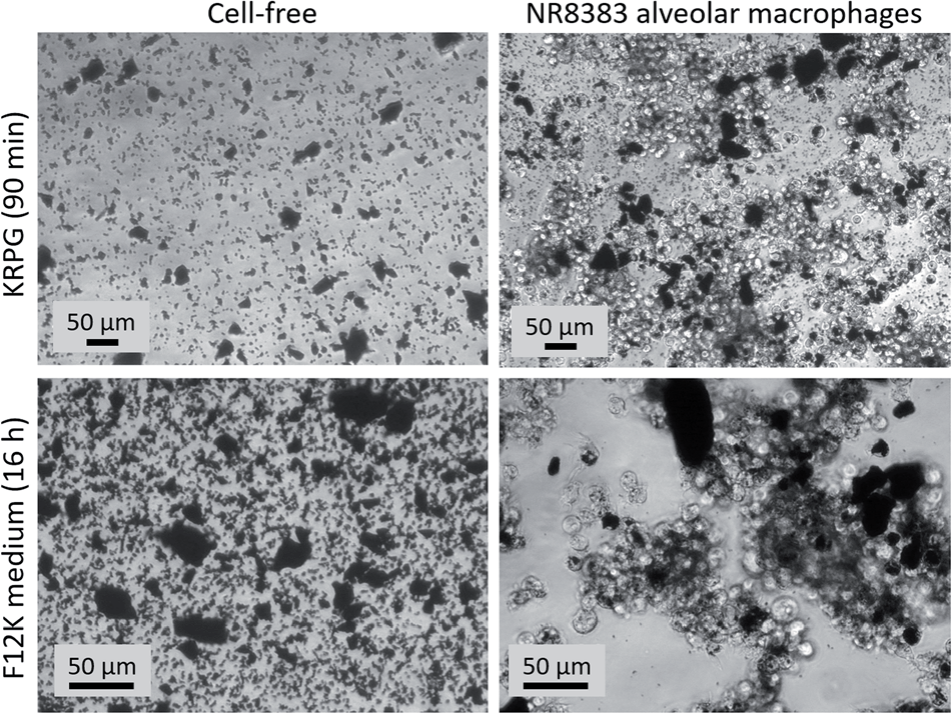
Sedimentation and uptake by alveolar macrophages of AC_06_01 (180 μg mL^−1^). Upper row: 90 min after adding particles in KRPG buffer and in the absence (left) or presence of cells (right). Lower row: 16 h after adding particles in F-12K medium and in the absence (left) and presence of cells (right). Some large particles were not phagocytized.

**Fig. 7:**
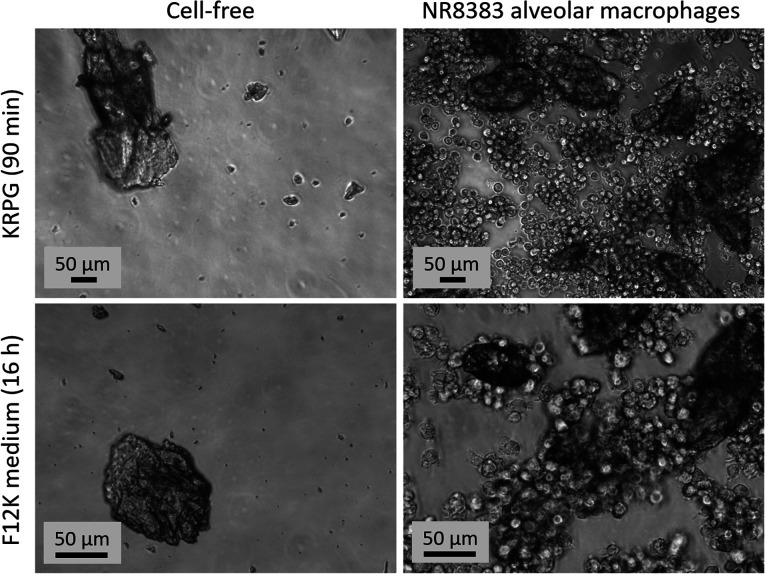
Sedimentation and uptake by alveolar macrophages of PU_02_02 (180 μg mL^−1^). Upper row: 90 min after adding particles in KRPG buffer in the absence (left) and presence of cells (right). Lower row: 16 h after adding particles in F-12K medium in the absence (left) and presence of cells (right). Cells are seen in close contact to the large particles.

Aerogel preparations mostly consisted of particles which were ingestible (<10 μm) for macrophages. Only the fraction with sufficiently small external dimensions were effectively ingested by the cells during the *in vitro* test, as was observed by phase contrast microscopy (Fig. S1 to S3†). However, due to the method of powder preparation, larger particles were administered as well (Fig. 6, 7, S1 and S2†). Macrophages may interact with these particles in different ways, which likely contribute to the outcome of the test. For instance, large particles may lead to frustrated phagocytosis.^50,51^ Aerogels may elicit specific surface reaction or release soluble substances, all of which may contribute to an activation or to the formation of cytokines.^52^

#### *In vitro* effects

**Fig. 8:**
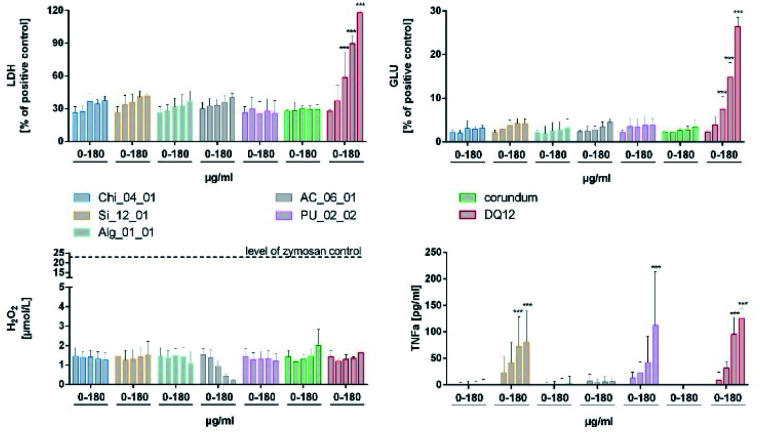
*In vitro* effects of aerogels on NR8383 cells. Cytotoxicity was indicated by activity of lactate dehydrogenase (LDH, upper left) and glucuronidase (GLU, upper right) and expressed relative to the 0.1% Triton-X100 positive control. Release of H_2_O_2_ measured with the Ampliflu Red method (lower left); the black dotted line indicates the level reached upon zymosan stimulation (positive control). Release of tumor necrosis factor α (TNFα, lower right) was measured with a specific ELISA. Corundum and quartz DQ12 were included as negative and positive controls, respectively. Data are presented as mean and standard error of the mean (***p < 0.001).

Cytotoxic and/or activating responses of aerogels were not detectable with respect to the release of LDH, GLU and H_2_O_2_ up to a concentration of 180 μg mL^−1^ (Fig. 8). However, the silicate Si_12_01, and the polyurethane PU_02_02 induced a dose-dependent formation of TNFα which became significant upon 90 and 180 μg mL^−1^, respectively. These two aerogels had to be tested in higher tier method to calibrate the *in vitro* findings for all other tested materials, or be removed from the candidate group.

In the frame of previous projects on nanoparticles, the NR8383 *in vitro* assay achieved a 95% accuracy to predict the assignment into either “active” or “passive” categories by *in vivo* inhalation testing.^48^ Nanoparticles were predicted to be “active”, if at least two of the four parameters (LDH, GLU, TNFα, H_2_O_2_) were significantly increased at a specific threshold (in metrics of surface area per macrophage). For aerogels, such *in vitro*–*in vivo* validation has not been performed yet. However, the TNFα induction of both, Si_12_01 and PU_02_0 were below the threshold, if the BET surface was used for calculation.

Interestingly, the most hydrophobic material (AC_06_01) induced a dose-dependent reduction of the H_2_O_2_ indicator to levels underscoring the untreated cell control. This may be due to an adsorption of the H_2_O_2_ indicator reagent (Amplex Red) and does not reflect a cellular mechanism. Similar sources of error have been reported for high-surface-area nanomaterials, such as hydrophobic MWCNTs.^53–55^ From this perspective, also the high FRAS activity of AC_06_01 might have been influenced by the adsorption of assay components. Of course, a substantial removal of essential biomolecules from the test system may falsify the results, which is one more reason to calibrate the Tier 1 abiotic and Tier 2 *in vitro* results by Tier 3 *in vivo* testing.

### Tier 3: *in vivo* effects of PU_02_02 aerogel

#### Selection of test material

ECHA recommends an “adequate choice of testing material”,^37,39^ in other words, testing the worst case of a group in higher-tier testing. Based on the absence of any biodissolution in Tier 1 (Fig. 3), the stability of the mesopores (Fig. 4), and the *in vitro* results of Tier 2 (Fig. 8), we selected the polyurethane aerogel PU_02_02 for this purpose. This polyurethane aerogel is a representative of the large candidate group of organic aerogels with low abiotic reactivity (Fig. 2), and absence of cytotoxicity in LDH, GLU and H_2_O_2_ indicators. Nevertheless, among other aerogels it stands out as worst case by its dose-dependent induction of TNFα (Fig. 8). The non-biological origin of PU as opposed to alginate, chitosan, or cellulose was not a formal criterion; also it did not contradict its selection as worst case.

#### Study design and control results

**Fig. 9:**
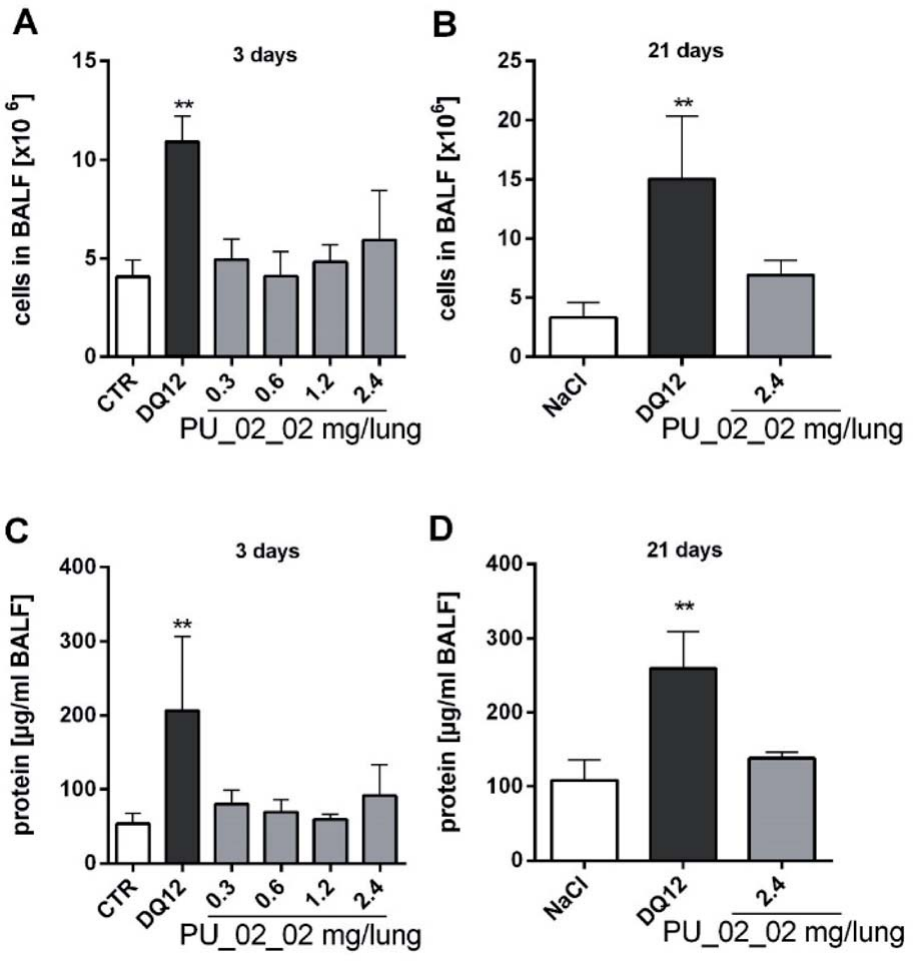
Cell counts and total protein concentration from broncho-alveolar lavage fluid (BALF) 3 d and 21 d post intratracheal instillation of PU_02_02 into rat lungs. Total cells (A) and (B) and total protein (C) and (D) after 3 (A) and (C), and 21 days (B) and (D). Doses of PU_02_02 administered per lung are indicated underneath in mg. Quartz DQ12 (1.2 mg per rat lung) and vehicle (0.5 mL of saline) were used as positive and negative controls, respectively. Mean values ± SD from *n* = 5 animals per group. **p < 0.01 (1way-ANOVA, post hoc Dunnett's multiple comparison test).

We performed an intratracheal instillation study with a respirable fraction of PU_02_02. Effects were compared to those of vehicle controls and quartz DQ12-treated animals (1.2 mg per lung) on day 3 and 21 post administration. Concentrations of PU_02_02 were 0.3, 0.6, 1.2, and 2.4 mg on day 3, and 2.4 mg per lung on day 21 (*n* = 5 animals per study group). Analyses of the broncho-alveolar fluid (BALF, see Fig. 9) showed the typical dominant population of alveolar macrophages (AM, viability > 95%), the absence of polymorphonuclear granulocytes (PMN), and a low concentration of total protein in vehicle-treated animals. As expected, quartz DQ12 led to progressively increasing total cell counts compared to control (day 3: 2.7-fold, day 21: 4.5-fold) with a prominent percentage of PMN (day 3: 27.4 ± 8.2%, day 21: 20.0 ± 2.2%), low numbers of lymphocytes (<0.5%), and increased protein concentration (4-fold). These results from control animals demonstrated the validity of the rat model.

#### Aerogel effects

**Fig. 10:**
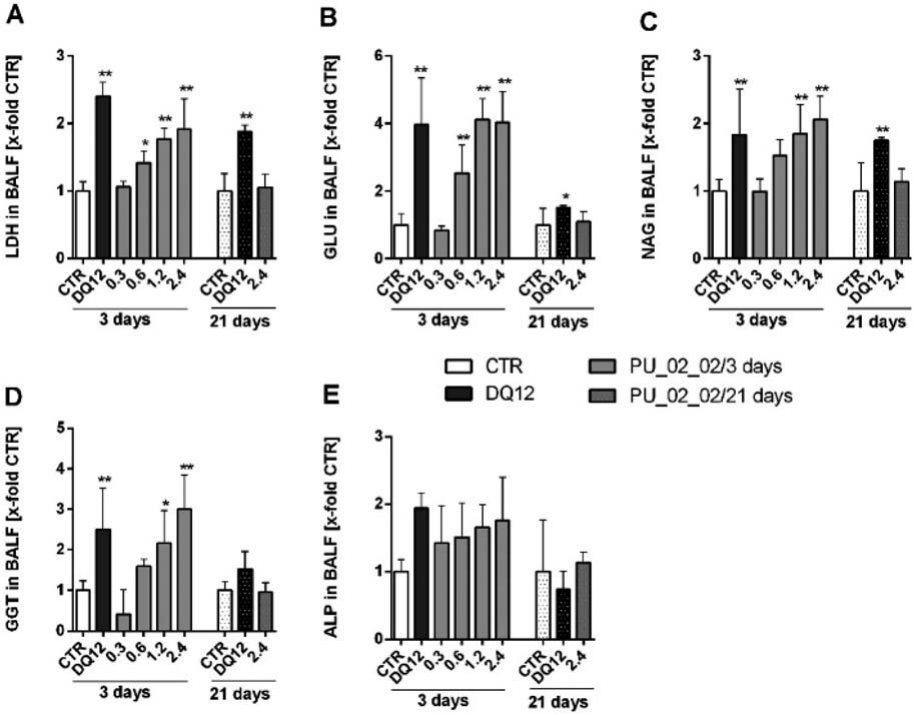
Enzyme activities in broncho-alveolar lavage fluid (BALF) 3 d and 21 d post intratracheal instillation of PU_02_02 into rat lungs. Values are shown in relation to the vehicle-treated control. Doses of PU_02_02 are given in mg per rat lung. Quartz DQ12 (1.2 mg per rat lung) and vehicle (0.5 mL of saline) were used as positive and negative control, respectively. (A) Lactate dehydrogenase, (B) glucuronidase (GLU), (C) *N*-acetylglucoseaminidase (NAG), (D) γ-glutamyl transferase (GGT), and (E) alkaline phosphatase (ALP). Mean values ± SD from *n* = 5 animals per group. **p < 0.01 (1way-ANOVA, post hoc Dunnett's multiple comparison test).

Administration of PU_02_02 did not evoke any clinical findings on days 3 and 21 (all study groups). Also, gross biopsy, animal and organ weight increases were unchanged, except for mediastinal lymph nodes which had increased in weight on day 21 (vehicle control: 21 ± 7 mg, quartz DQ12: 63 ± 11 mg, PU_02_02: 69 ± 26 mg). In the BALF, PU_02_02 elicited small, non-significant increases in cell numbers and protein concentration. Alveolar macrophages, many of which contained stained inclusions on day 3 and 21 (Fig. S4†), still formed the major fraction of cells. PMN counts were increased to 4% and 7% in 2/5 animals of the 2.4 mg PU_02_02 group only on day 3, but not on day 21, indicating a very mild and transient inflammatory reaction in the lung of these animals. A slightly decreased viability of BALF cells to 75% was accompanied by the occurrence of fine granular eosinophilic material (Fig. S1†). Interestingly, PU_02_02 treatment did not increase TNFα to detectable levels on day 3 (not shown) but led to dose-dependently increased enzyme activities of LDH, GLU, NAG, and GGT (Fig. 10), all of which are indicative for a cell and/or lysosomal damage. These effects became significant upon ≥0.6 mg PU_02_02 for LDH and GLU, and upon ≥1.2 mg for NAG and GGT, whereas ALP, which is indicative of damaged type-2 epithelial cells, showed no significant elevation (Fig. 10). All enzyme activities had decreased down to control levels on day 21, and this was in sharp contrast to the well-known pro-fibrotic effects of quartz DQ12.

#### Histopathology

The histopathological inspection of PU_02_02 treated lungs (Fig. S5†) revealed alveolar foreign material in all dose groups (0.3–2.4 mg), and confirmed the minimal to slight granulomatous broncho-interstitial inflammation, accompanied by a minimal to moderate mixed cell infiltration with dose-dependent severity on day 3. Starting at 0.6 mg per lung, minimal to moderate alveolar histiocytosis, hypertrophy/hyperplasia of terminal bronchioles, and an alveolar lipoproteinosis were noted. On day 21, foreign material was still present in macrophages, and all signs of a slight impairment (alveolar histiocytosis, mixed cell infiltration and granulomatous broncho-interstitial inflammation) were still detectable. Together these results show that the aerogel PU_02_02 particles in the lung have a very low toxicity and may elicit a mild transient inflammation if the instilled lung burden is equal to 0.6 mg or larger.

As PU_02_02 showed no cytotoxicity in the alveolar macrophage assay (Fig. 8), its presence in the lung was expected to be well tolerated, although PU_02_02 induced some TNFα formation of NR8383 alveolar macrophages (Fig. 8). The mechanism behind this early TNFα induction is not yet clear but similar findings have been reported also for J774 mouse macrophages subjected to polyurethane particles.^56^ Similarly amorphous and crystalline silica particles induce TNFα formation of macrophages.^57^ In line with this, the *in vivo* study, carried out with a sub-fraction of smaller PU_02_02 particles than used *in vitro*, revealed signs of a mild, transient inflammation, although no elevated levels of TNFα were found in BALF on day 3. Instead, elevated enzyme levels, which may have originated from a damaged macrophage population (on day 3 only) were most likely caused by an initial lung overload. These effects were confined to doses ≥0.6 mg per rat lung (Fig. S5†), which is roughly equivalent to a mean particle burden of 30–60 pg per AM (for calculation see^48^). Due to the density of PU_02_02 particles of approximately 1.03 g cm^−3^ (conservative estimation) this would be equivalent to a mean volume load of 29–58 μL, which is 3.2–6.4% the volume of an AM (900 μm^3^), a value known to impair AM function and particle clearance.^58,59^ However, as particles are likely bearing air inclusions and because their distribution throughout the lung is inhomogeneous due to the administration *via* instillation, the volume load for AM may be far higher in those regions of the lung which exhibited signs of hypercellularity and inflammation on day 21. However, as markers of epithelial cell damage such as ALP were not increased, and because BALF enzyme parameters returned to normal even at the highest dose of 2.4 mg on day 21, PU_02_02 particles in the lung parenchyma seem to be tolerated quite well. Together, this led us to suggest that PU_02_02 particles have a low toxic potential in the lung.

### Tier 2 and Tier 3: comparison to published toxicity of aerogels

Aerogels are attractive candidates for tissue engineering, regenerative medicine and other biomedical applications,^60^ and previous *in vitro* studies have focussed on these tempting possibilities. For example, RAW 264.7 macrophages were used to identify alginate as a non-toxic carrier for anti-inflammatory or antibacterial purposes.^31^ Similarly, aerogels of alginate, alginate-lignin or alginate-starch were investigated and found to be not toxic for fibroblasts or other cells.^30,33,61,62^ Also a silica-gelatin aerogel designed for the controlled release of the anti-cancer drug methotroxate was non-cytotoxic for HaCaT or HL60 cells.^63^ A polyurea-encapsulated silica aerogel, which is pursued as an implant material, was shown to be biocompatible towards blood constituents and vascular endothelial cells.^64^ However, apart from these biomedically motivated studies, cell culture tests with a more standardized protocol have not yet been conducted. We, therefore, suggested the NR8383 alveolar macrophage test as a versatile tool to screen aerogels for their bioactivity and prospective lung toxicity.

Also the few *in vivo* studies on aerogels were hitherto focussed on biomedical applications (*cf.* García-González *et al.* 2019).^28^ For example, aerogel microparticles based on silica, starch and sodium alginate were administered *via* gavage to investigate their drug releasing properties.^60^ Subcutaneous or intramuscular implantations of silica aerogels were employed to show that these aerogels elicited no local inflammation in rats and may be used as scaffolds for cell growth.^29^ With respect to the lung, systematic or even guideline-directed studies on aerogels have not yet been published. However, one explorative instillation study with a high dose of alginate-chitosan aerogel (35 mg kg^−1^) revealed acute hazardous effects in the rat lung, reflected by hypercellularity and thickening of alveolar walls.^32^ Considering that PU_02_02 elicited a mild transient inflammation and specific enzyme increases upon ≥0.6 mg per lung, such effects are conceivable but may be due to an overload of the lung. It should be stressed that instillation studies in contrast to inhalation studies are not covered by any OECD guideline. Nevertheless, they may be effective and versatile tools to investigate effects of Tier 2-preselected aerogels especially if the availability of respirable-size testing material is limited, as was the case for the 5 μm-filtered fraction of PU_02_02 particles.

Effects of aerogels in Tier 2 and 3 were moderate and/or reversible, but this is not enough to settle the question if respirable aerogel dusts may pose a health risk on humans. While the occupational and epidemiological^65^ aspect deserves attention especially for insulator materials, the method combination proposed in Tier 1–3 may help to identify critical materials.

### Category approaches

Considering the Tier 1 and Tier 2 identification of PU_02_02 as worst case among the candidate group of organic aerogels, considering the *in vivo* finding that PU_02_02 particles have a low toxic potential in the lung, our data can justify a category “polymer-based aerogels with low toxic potential”:

• Alg_01_01, Alg_01_02.

• AC_06_01.

• Cell_07_03, Cell_07_04.

• PU_02_03, PU_02_01, PU_02_02.

• Chi_04_10, Chi_04_11, Chi_04_01, Chi_04_13, Chi_04_06, Chi_04_07, Chi_04_08.

The above materials are similar in their Tier 1 and Tier 2 properties, and were assessed by the worst case in Tier 3. At least the alginate aerogels lose their nanoscale properties in relevant fluids, further lowering concerns. Based on insufficient similarity with the above group in the Tier 1 and Tier 2 findings, several materials had to be excluded from grouping:

• Silicates, because Si_12_01 induced TNFα *in vitro* (Fig. 8).

• Chitosan Chi_04_12 and the pyrolyzed carbon PA_12_01, because of a ≥10-fold higher abiotic reactivity than the others (Fig. 2).

We excluded all inorganic and non-organic carbonaceous materials from the polymer-based aerogel category stated above, but they might still be grouped with aerogels of similar compositions: the two silica-based aerogels were similar in Tier 1 and showed limited reactivity and significant dissolution. Therefore, (if investigated in detail) it might be justifiable to gather them within one group or category of aerogels of limited biodurability, which might be assessed with existing *in vivo* data.^22–26^ With respect to the non-organic carbonaceous materials we suggest not to combine charcoal (derived from coconut shell) in one group with pyrolyzed carbonaceous aerogels because of large differences in reactivity.

## Conclusions

Aerogels are an example of advanced materials whose functionality in the intended use is enabled by properties on the nanoscale (the open internal porosity), but which do not fulfill the REACH definition of a nanomaterial. Aerogels do not require registration as nanoforms, but their nanostructures raise concerns which need to be addressed. To assess the possible hazards of organic aerogels, we derived a tiered testing strategy (Fig. 1) from current frameworks for the grouping and testing of nanomaterials: Tier 1 and Tier 2 encompass biophysical and *in vitro* toxicity screenings and allow to identify sufficiently similar candidate materials in order to define distinct categories of aerogels. Materials with obviously deviating properties were removed from the category and are intended for an individual assessment. These outlying cases were one carbonaceous (pyrolysed) aerogel, the silicate aerogels, and one of the eight chitosan aerogels. Groups of aerogels with similar chemical composition such as the group of chitosan compounds, the silicate group, the alginate group, as well as the cellulose group, show similar dissolution and reactivity within their group. Based on its lacking biodissolution in Tier 1 (Fig. 3), the stability of its mesopores (Fig. 4), and the *in vitro* results of Tier 2 (Fig. 8), we chose polyurethane aerogel as an adequate testing material to further assess asses its bioactivity *in vivo* (Tier 3). Although the acute induction of TNFα induced by polyurethane in alveolar macrophages (Tier 2) was not detected in the lung lavage 3 days post intratracheal administration, there were other effects pointing to a moderate and reversible cell damage. Thus, organic aerogels, as tested here, may be understood as a category “polymer-based aerogels with low toxic potential”.

The properties recommended by the ECHA guidance for grouping of nanomaterials,^37^ and its implementation with specific methods by GRACIOUS,^40^ DF4nanogrouping^42^ or nanoGRAVUR^41^ provide a tiered framework to assess concerns about the content (and potential release) of nanostructures in advanced materials. For more complex advanced materials, one would first assess the form of release, which may have internal or external nanostructures, and more than one chemical component. This step was omitted here due to monoconstituent materials, but it may become important to add a layer of similarity assessment with respect to the form and the rate of release, as established by the NanoRelease stepwise decision framework (ISO TR 22293, to be published 2021). To demonstrate safe use, beyond regulatory requirements, categorization across different chemical substances can then be based on the similarity of physical structure and inherent toxicity of the components (selected from Tier 1 of the nanoGRAVUR framework), substantiated further by abiotic and *in vitro* reactivity testing (Tier 2). Here we supported the validity of the approach by selective *in vivo* testing (Tier 3), which reduces the safety margins of the assessment, but should not be needed in all cases.^38,66^ Other classes of advanced materials may have no nano-specific concerns at all,^11,12^ and might instead require assessment of microplastic properties or assessment of leaching of small organic additives or metals. The present approach would not be applicable then. Our approach exemplifies how nanosafety concepts, and methods developed on particles, can be applied to handle specific concerns about advanced materials, *i.e.* materials which contain or release nanostructures, even if they may not contain nanoparticles.^67^ Future applications of that approach might explore cementitious systems,^68^ superconductive cables, water purification systems,^69–71^ and even more.^72^

## Experimental

### Preparation of aerogel materials

The tests were conducted on a broad set of materials, consisting of mostly organic aerogels made from alginate, chitosan, cellulose, carbon, polyurethane and silicate (Table 1). Partners providing these materials were German Aerospace Center (DLR), Dräger, Technical University Hamburg-Harburg (TUHH), Center for Materials Forming (CEMEF) of MINES ParisTech/ARMINES, National and Kapodistrian University of Athens (NKUA) and BASF. The organic aerogels are characterised by a porosity between 70% and 90%. The preparation procedure of the aerogels is listed in the ESI.†

### Tier 1 (abiotic screening) methods

#### Ferric reduction ability of serum (FRAS)

The method and handling follows the SOP in.^73^ The working principle of the FRAS assay is based on the oxidative damage on antioxidants present in human blood serum (HBS).^44–46^ Human blood serum is incubated together with the oxidizing material such as the aerogels at a temperature of 37 °C for 3 h, inducing a damage to the antioxidants naturally present in HBS, *e.g.* Vitamin E. The particles are removed by centrifugation, and HBS is pipetted into a solution of complexed Fe^3+^, where the remaining antioxidants reduce Fe^3+^ to Fe^2+^. This change in color is detected *via* UV-VIS spectroscopy (Lambda 35, Perkin Elmer). The oxidative damage is considered significant if triplicate error bars are not consistent with the negative control.

#### Static dissolution of aerogels

As a Tier 1 testing the method, the dissolution of aerogels was investigated under static conditions^74^ in two relevant physiological media, phagolysosomal simulant fluid (PSF) with pH 4.5 (Table S1†) simulating the acidic conditions particles are exposed to after uptake by alveolar macrophages^75^ and gastric intestinal simulating fluid (GIF)^76^ at pH 1.6 (Table S2†). Previously, transformation of crystalline nano-cellulose was assessed in the same lysosomal fluid PSF with XRD detection of structural transformations, but the dissolved content was not analysed.^77^ To minimize the organic background of the media, organic compounds and organic acids were omitted from the media. The organic acids can act as metal chelating complexes, and thus have limited impact on polymer dissolution. 10 mg of aerogel were weighted into a 100 mL Duran® borosilicate glass flask and 80 mL of simulating fluid were added. The flasks were then placed in an orbital shaker from Vibrax-VXR from Ika in a heating chamber with a temperature of 37 °C and continuously shaked for 24 h. After the incubation time, 30 mL of each flask were filtered through a 1 μm glassfibre filter, followed by a 0.02 μm alumina filter to achieve a size cutoff of 20 nm. The total organic carbon (TOC) was then analyzed with a TOC-L from Shimadzu in triplicates and an accuracy of 1 mg L^−1^. The sample was completely combusted in the TOC-L at a temperature of 680 °C in an oxygen rich environment. The amount of CO_2_ was then detected through a nondispersive infrared detector.

### Tier 2 (*in vitro*) method

#### Preparation of particle suspensions

Details of the procedure have been published.^48^ For each round, powder materials as provided by the consortium members were freshly suspended in KRPG buffer (see below) or F-12K medium using ultrasonication with a 3 mm probe (VibraCell, Sonics & Materials, 50 W) for 10 s. The suspensions prepared this way contained 360 μg per mL KRPG buffer (for H_2_O_2_ production measurements) or 180 μg mL^−1^ F-12K medium (for cell culture testing). Final concentrations of 180, 90 45 and 22.5 μg mL^−1^ were achieved by serial dilution in the respective medium.

#### Cells and culture conditions

The alveolar macrophage cell line NR8383 (ATCC) was used and cultured according to ATCC guidelines in Ham's F12K (Kaighn's modification) supplemented with l-glutamine, 15% (v/v) and heat inactivated fetal bovine serum (PAN Biotech), and penicillin/streptomycin (PAN Biotech). Composition of KRPG-buffer was (in mM): NaCl (129 mM), KCl (4.86 mM), CaCl_2_ (1.22 mM), NaH_2_PO4 (15.8 mM), glucose (5.5 mM), pH 7.3–7.4.

#### NR8383 alveolar macrophage *in vitro* assay

Previous studies have shown that adverse or inflammatory effects of respirable (nano)particles in the lung can be determined with the NR8383 alveolar macrophage assay.^48^ In that assay the release into the cell culture medium of lactate dehydrogenase (LDH), of the lytic enzyme glucuronidase (GLU), of H_2_O_2_ (spontaneous ROS-formation), and of tumor necrosis factor α (TNFα), is tested. These parameters describe different modes of cell damage and/or biological response of alveolar macrophages (AM) with relevance for *in vivo* toxicity. Thus, the release of LDH reflects membrane damage and/or necrosis of AM. Glucuronidase is a representative of lytic enzymes which may be released during activation of macrophages from lysosomes. TNF is an important pro-inflammatory cytokine produced, among others, by alveolar macrophages. The formation of H_2_O_2_ by macrophages is induced by pathogens such as yeast, but also by particles. Measurement of extracellular H_2_O_2_ indicates the oxidative potential particle-laden macrophages may have for neighboring cells. Particle effects are bench-marked under serum-free conditions against quartz DQ12, a well-accepted pro-inflammatory and fibrogenic positive control, and corundum, which elicits no such effects in the lung.^48^

Cell culture assays were carried out in 96 well plates using 4 concentrations of particles (triplicates) which were pipetted onto NR8383 cells (3 × 10^5^ cell/well) under serum-free conditions and incubated for 16 h.^48^ In brief, cell culture supernatants were tested for LDH, GLU and TNFα activity (in triplicates). Controls included untreated cells (CTR), triton X-100-treated cells (to fully release LDH and GLU), and lipopolysaccharide (LPS)-treated cells to test for the macrophages' TNFα production ability. Particle-free controls were run side-by-side for each particle concentration and were used to correct for light scattering properties. LDH was tested with Roche Cytotoxicity Detection Kit. GLU activity was measured using *p*-nitrophenyl-β-d-glucuronide as a substrate. TNFα was tested using a dedicated enzyme-linked immunosorbent assay (ELISA) specific for rat TNF (Bio-Techne, Wiesbaden, Germany). H_2_O_2_ release was measured in KRPG buffer using the Amplex Red reagent after a 90 min particle exposure. Correctness of the photometric determination of H_2_O_2_ concentration was tested with a fixed concentration of H_2_O_2_ (30 μM). Competence of the NR8383 cells to produce H_2_O_2_ was controlled with zymosan stimulation. Photometric analysis of 96 well plates were carried out with a Tecan Infinite F200 Pro plate reader (Tecan GmbH, Crailsheim, Germany). Cell cultures, as well as particle sedimentation and uptake were micrographed with an inverted phase contrast microscope (Zeiss Axiovert C40) equipped with an AxioCam II Camera and AxioVison software.

### Tier 3 (*in vivo*) method

#### For intratracheal instillation

Larger non-respirable particles had to be removed from the suspension by filtering as described.^49,57^ Therefore, PU_02_02 was dispersed in pyrogen-free H_2_O (B. Braun Melsungen AG, Germany), ultrasonicated as described for *in vitro* studies, and gravity-passed through sterile 5 μm filter gauze (Sysmex Partec GmbH, Görlitz, Germany). Fluid containing the effluent particle fraction was frozen at −20 °C, lyophilized, re-suspended in saline (0.9% NaCl), and adjusted to the maximum concentration of 4.8 mg mL^−1^. Particle suspensions (0.5 mL) were intratracheally administered (5 animals per group) to pathogen-free female Wistar rats (200–250 g, strain WU, Charles River Laboratories, Sulzfeld, Germany) under deep isoflurane anesthesia. Quartz DQ12 (1.2 mg) and saline were used as positive and negative control, respectively. Animal experiments were carried out at the University Clinics of Essen, Germany, and were ethically approved by local authorities (LANUV, Recklinghausen, Germany, Accession no. 84-02.04.2011.A157).

#### BALF analysis

Rats were deeply anaesthetized with a mixture of ketamine and xylazine administered intraperitoneally. Citrate blood was retrieved from the left ventricle to prepare hemograms using a Sysmex KX-21N instrument (Sysmex Europe GmbH, Norderstedt, Germany) and blood smears. Broncho-alveolar lavage fluid (BALF) was collected by washing the right lung (left bronchus clamped) with saline (5 × 3 mL). After the second wash, 1 mL BALF was retrieved from the pooled BALF to measure enzyme activities. After three further washes, pooled BALF were stored at 4 °C. Lavaged cells were pelleted (100 g, 10 min), washed with phosphate buffered saline, re-suspended in saline and counted with a Coulter Counter Z2 (Beckmann Coulter GmbH, Krefeld, Germany). Cell viability was determined *via* trypan blue exclusion in a Neubauer chamber. Cytospin preparations of pelleted cells (1000 g, 4 °C, 10 min) were stained with May-Grünewald/Giemsa dyes according to standard protocols. The BALF supernatant was centrifuged (1800 g, 4 °C, 10 min) and aliquots were stored frozen to measure total protein (Lowry method), or TNFα (cytolysis test with L929 fibroblasts).^78^ Lactate dehydrogenase (LDH) and glucuronidase activity (GLU) was measured as described above for cell culture supernatant. To measure *N*-acetylglucosaminidase (NAG) 10 μL of the BALF supernatant were mixed with 40 μL citrate buffer (100 mM sodium citrate, 0.02% albumin fraction V, pH 5) and 50 μL substrate solution (10 mM 4-nitrophenyl *N*-acetyl-β-d-glucosaminide). Incubation at 37 °C was stopped with 100 μL 4% NaOH after 4 h. Optical density was measured at 405 nm against 0.9% NaCl. Alkaline phosphatase (ALP) and γ-glutamyl transferase (GGT) were measured using a fully automated ADVIA 1800 Siemens System (Siemens Healthcare GmbH, Erlangen Germany).

#### Preparation for histopathology

After complete withdrawal of BALF from the right lung (Fig. S5†), the clamp closing the left bronchus was removed and the right lung was inflated with 4% formaldehyde fixative (pressure: 30 cm H_2_O). Lungs were embedded in paraffin for routine pathology, which was conducted at BASF-SE according to standard protocols. As the lavage procedure of the right lung had no major impact on histopathological staging, the observations from both lung lobes were combined (Fig. S5†).

#### Statistics

All *in vitro* assays were performed in three independent experiments with triplicates being used in the cell cultures. Data represent mean ± standard deviation (SD). For the *in vivo* studies, mean ± SD was calculated from 5 animals per group. *In vitro* and *in vivo* data were compared pair-wise to the corresponding control group by two-way ANOVA and post-hoc Dunnett's multiple comparison test, using Graph Pad Prism software (version 6). A value of *p* ≤ 0.05/*p* ≤ 0.01 was considered significant (*/**).

## Ethics approval

Animal experiments were carried out at the University Clinics of Essen, Germany, and were ethically approved by local authorities (LANUV, Recklinghausen, Germany, Accession no. 84-02.04.2011.A157)

## Author contributions

Conceptualization: WW, MW; investigation: AV, JGK, SG; methodology: KW; supervision: RL; writing – original draft: JGK, MW, WW, SG; writing – review & editing: MW, WW.

## Conflicts of interest

JK, SG, KW, RL and WW are employees of BASF SE, a company producing nanomaterials. All other authors declare that they have no competing interests.

## Supplementary Material

NA-003-D1NA00044F-s001
