# Supplementary material for: Aerogels are not regulated as nanomaterials, but can be assessed by tiered testing and grouping strategies for nanomaterials
Source: Nanoscale Adv. 2021 May 19;3(13):3881–93. doi: 10.1039/d1na00044f (PMC9419173; doi:10.1039/d1na00044f)
Supplement: NA-003-D1NA00044F-s001 [file NA-003-D1NA00044F-s001.pdf]

Electronic Supplementary Material (ESI) for Nanoscale Advances.
This journal is © The Royal Society of Chemistry 2021

Electronic Supplementary Information

Aerogels are not regulated as nanomaterials, but can be assessed by tiered testing and grouping strategies for nanomaterials

Johannes G. Keller[a†], Martin Wiemann[b†], Sibylle Gröters[a], Kai Werle[a], Antje Vennemann[b], Robert Landsiedel[a], Wendel Wohlleben[a*]

Preparation Procedure of Aerogels

All Chitosan aerogels were produced by DLR as follows: Chi_04_10 is a poly(propylene-glycol)diglycidyl ether(PPGDGE)-functionalized chitosan aerogel and was produced by creating a 3% chitosan solution in 1:1 wt% water and ethanol mixture and 3% acetic acid. After mixing with PPGDGE at 2-8 °C, the solution was stirred for 15 minutes at room temperature. Then the mixture was heated to 50 °C for 90 minutes. After cooling down the mixture, using a syringe dropping technique, the droplets of the chitosan solution was treated with 8 wt% of sodium hydroxide in a mixture of ethanol and water (1:1 wt%). Then the wet gel beads were prepared: After washing several times with water and a solvent exchange against ethanol, the alcogels were subjected to supercritical drying. Finally, the chitosan aerogel beds were powdered.

In more detail, the monolithic form of PPGDGE-functionalized chitosan aerogel Chi_04_11 was produced the same way as Chi_04_10 but was heated at 40 °C for 48 hours instead of 50 °C for 90 minutes. After cooling down the mixture, the solution was layered with 8 wt% of sodium hydroxide solution in a mixture of ethanol and water (1:1 wt%) to induce gel formation. After the wet gel in monolithic form was prepared, several washing steps with water and a solvent exchange against ethanol were carried out. The alcogels were then subjected to supercritical drying. Chi_04_01 is a poly(ethylene glycol) diglycidyl ether (PEGDGE)-functionalized chitosan aerogel and is produced by preparing a homogeneous clear solution of 3 g chitosan (purchased from Glentham Life Scienes Ltd.) in 97 mL of aqueous acetic acid solution (3 wt%). The viscous solution was mixed with PEGDGE at 2-8 °C. And further stirred at room temperature for 30 minutes. After heating the homogeneous mixture at 80 °C for 90 minutes, the solution was cooled to room temperature. The pH of the final solution was raised to 4.5. After cooling down the mixture, using syringe dropping technique, the droplets of the chitosan solution were treated with 8 wt% of sodium hydroxide solution in water. The wet gel beads were prepared. After washing several times with water followed by solvent exchange with ethanol, the alcogels were subjected to supercritical drying. The two monolithic forms of ureido-functionalized chitosan aerogel, Chi_04_13 and Chi_04_12 were prepared according to the reported procedure in Literature.[1] For the toxicological testing, all aerogels were powdered with a mortar. Chi_04_13: Chitosan solution (2 wt%) was prepared by dissolving chitosan powder in aqueous acetic acid in the presence of urea. The molar concentration ratio between acetic acid and urea was 1:6. After purging nitrogen gas through the chitosan solution, it was poured into a mold and heated at 70°C until the solution turned into a gel (about 16 h). During gelation the pH of the reaction mixture was raised from pH 3.2 to pH 5.4. The gel media was washed with aqueous NaOH solution, several times with water and finally with ethanol. After supercritical drying, the monolithic form of aerogels was obtained. Chi_04_12 was prepared by following the same procedure using hydrochloric acid instead of acetic

acid for the preparation of the chitosan solution. In this case, the gelation occurred after 36 hours at 70°C. After gelation, the wet gel body was washed with aqueous NaOH solution, washed with water and finally with ethanol. After supercritical drying, the monolithic form of aerogels was obtained. Chi_04_06 was used as the reference sample(not an aerogel) and consisted of pure chitosan powder bought from Glentham Life Sciences Ltd. GP7325. Degree of deacetylation: >90 %. Chi_04_07 was used as the aerogel reference sample which was prepared by mixing 40 g of chitosan with 920 mL water. Then 40 g of glacial acidic acid were added and vigorously stirred until the solution became homogeneous (approx. 60 min). A Jet-Cutter was used for bead preparation. The regeneration bath was of 2.5 wt% sodium hydroxide. Beads were sieved after full gelation. Rinsing was performed with deionized water. Stepwise solvent exchange with 99% ethanol was performed prior to supercritical drying with CO_2 (115 bar, 60 °C). Mortar and pestle were used for manual powder preparation. Chi_04_08 is a polyethyleneimine functionalized chitosan aerogel. The beads were prepared identically to Chi_04_07 followed by a two-step functionalization. Gel bead preparation was identical with Chi_04_07. Functionalization was performed in two steps. Step 1: 8.57 mL epichlorhydrin was added to 500 mL chitosan gel beads and 500 mL ethanol and stirred at 50°C and 60 min. Step 2: Addition of 50 mL water with 4.2 g NaOH. Addition of 21.86 g polyethyleneimine (Sigma-Aldrich, Mw=800g/mol, Mn= 600g/mol, product code: 408719). 60°C and 60 min stirring. Excess of ammonia was added followed by washing with ethanol. The supercritical drying was conducted identically to the preparation procedure of Chi_04_07. For the generation of powder, mortar and pestle was used.

BASF produced PU aerogels by reacting isocyanate and a crosslinking composition in methylethylketone at room temperature followed by drying of wet-gels with supercritical CO_2.[2]

Innventia-RISE produced the cellulose pulp and CEMEF made Aerogel beads Cell_07_03 and Cell_07_04 through ethanol and HCl coagulation.[1] The pulp was dissolved in 8% NaOH(aq) solution[3] processed with the Jet-Cutter and coagulated in ethanol (Cell_07_03) or in 1 M HCl (Cell_07_04). The obtained cellulose coagulated particles were then washed with ethanol and dried under CO_2 supercritical conditions resulting in aerogel beads with a mean diameter of 0.6 ± 0.16 mm. The difference between both aerogels is based on the non-solvent used for coagulation. The samples consist of 97.1% cellulose, 0.8% lignin, 2.1% hemicellulose.

The National and Kapodistrian University of Athens (NKUA) produced the pyrolyzed carbon PA_12_01 from polyamide aerogel pellets which were synthesized from the reaction of Desmodur RE (from Covestro AG) and pyromellitic acid in tetrahydrofuran at room temperature and subsequent drying of wet-gels with SCF CO_2 according to a modification of literature procedures concerning the preparation of aerogel monoliths.[4] Afterwards the pellets were pyrolyzed in a tube furnace at 800 °C for 5 h under flowing argon and subsequently at 1000 °C for another 3 h under flowing CO_2. All flow-rates were set to 150 mL min^{-1}.

Furthermore, NKUA produced both Si_12_01 and Si_12_02 following a modification of Zhang et al. 2004 procedure.[5] Silica Aerogels were synthesized from the reaction of tetramethyl orthosilicate (Acros) and ammonium hydroxide in methanol and subsequent drying of wet-gels with liquid CO_2 in an autoclave under subcritical conditions (20 °C) for Si_12_01, whereas Si_12_02 wet-gels were dried under ambient conditions for one week.

AC_06_01, the activated carbon was produced by Draeger. The commercially raw material of coconut shell with a grain size 12x20 mm was grinded to <100 µm. The material consists of > 95 m% C, < 2 m% K and < 2 m% O.

The two Alginate aerogels, Alg_01_01 and Alg_01_02 were produced by dissolving sodium alginate in deionized water and $CaCO_3$ powder dispersed into it. For Alg_01_01 the jet cutter was used as a

processing method. Sodium alginate was dissolved in deionized water and loaded into the feeding vessel of the jet -cutter. Pressurized air was used to pressurize the vessel and push the alginate solution though the nozzle at the required velocity. The alginate droplets formed by the cutting action of the jet cutter were collected in a $CaCl_2$ bath where the gelation took place. They were kept in the gelation bath for 5 to 10 min before being removed and transferred into pure ethanol for the solvent exchange yielding a particle size of 700 µm. In the production process of Alg_01_02 the coarse emulsion was produced by dispersing the alginate solution in rapeseed oil at a low stirring rate (160RPM) for 10 min before processing it in one pass through the colloid-mill. The gelation trigger was prepared beforehand by dispersing 50 wt% acetic acid solution in rapeseed oil where Span80 surfactant was previously dissolved in (2wt% respective of the oil mass). It was then added to the alginate emulsion at the output of the colloid-mill and the resulting emulsion/suspension was stirred at 160 RPM for 1h to ensure complete gelation. The gel micro-particles were separated from the oil by adding a 30 wt% ethanol solution to the suspension and let to sediment overnight. Once sedimented, the particles were at the bottom of the vessel in the ethanol solution. One further washing step was done with 30 wt% ethanol before further continuing the solvent exchange with pure ethanol until the ethanol concentration in the gel micro-particles was at least 96 wt%. The supercritical drying was carried at 60°C and 120 bar for 2h with a continuous flow of CO2 of about 20 g/min. With a particle size of d_{32} = 15 µm and d_{v90} = 35 µm.

Table S1 Composition of PSF pH4.5 lysosomal simulant fluid

**PSF[6, 7] without organic compounds**		**[mg/L]**	**[g]**
**Sodium phosphate dibasic anhydrous**	Na_2HPO_4	171	0.855
**Sodium chloride**	NaCl	6650	33.250
**Sodium sulfate anhydrous**	Na_2SO_4	71	0.355
**Calcium chloride dehydrate**	$CaCl_2x2H_2O$	29	0.145
**Hydrochloride acid for pH 4.5**	HCl		
**Ultrapure water**			

Table S2 Composition of GIF pH1.6 stomach simulant fluid

**FaSSGF[8]** without organic compounds		**[mM]**
**Sodium chloride**	NaCl	34.2
**Hydrochloride acid for pH 1.6**	HCl	
**Ultrapure water**		

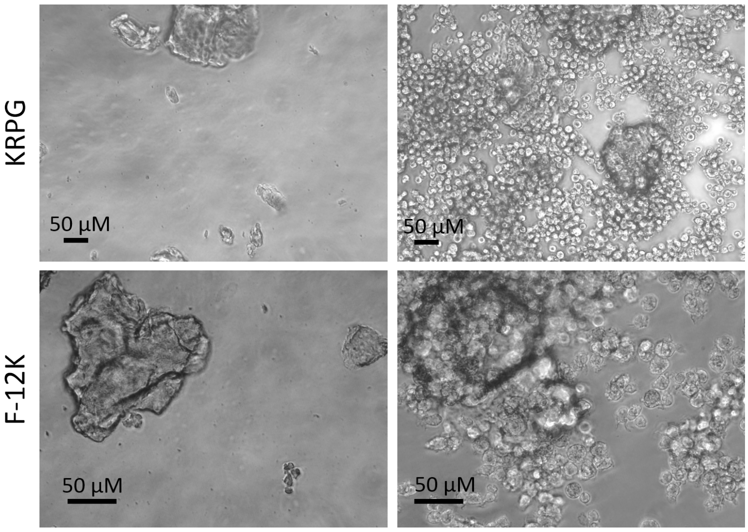

Figure S1 Sedimentation and uptake of **Chi_04_01** (180 µg/mL). Upper row: In KRPG buffer in the absence (left) and presence of cells (right) after 90 min after addition of particles. Lower row: In F-12K medium in the absence (left) and presence of cells (right) after 16 h after addition of particles. Note that cells in either solution interact with large particles. Few small particles visible under cell-free conditions and may have been internalized. Also, with this treatment cells appear healthy.

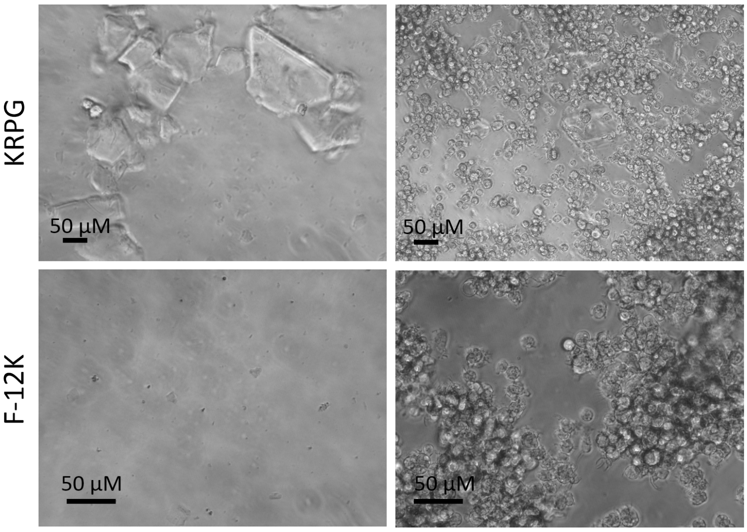

Figure S2 Sedimentation and uptake of **Si_12_01** (180 µg/mL). Upper row: In KRPG buffer in the absence (left) and presence of cells (right) after 90 min after addition of particles. Lower row: In F-12K medium in the absence (left) and presence of cells (right) after 16 h after addition of particles. Note that particles are large and slab-like and hardly interact with the cells which show no signs of damage.

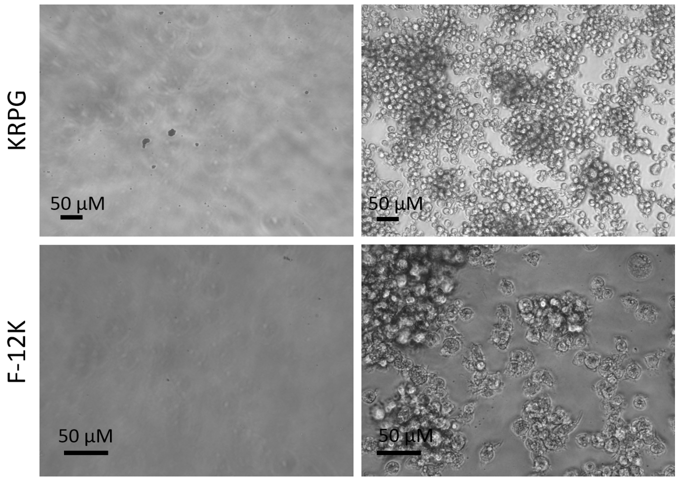

Figure S3 Sedimentation and uptake of **ALG_01_01** (180 µg/mL). Upper row: In KRPG buffer in the absence (left) and presence of cells (right) after 90 min after addition of particles. Lower row: In F-12K medium in the absence (left) and presence of cells (right) after 16 h after addition of particles. Note that alginate spheres are not visible in the focal plane of the cells and do not interact with the cells which appear healthy.

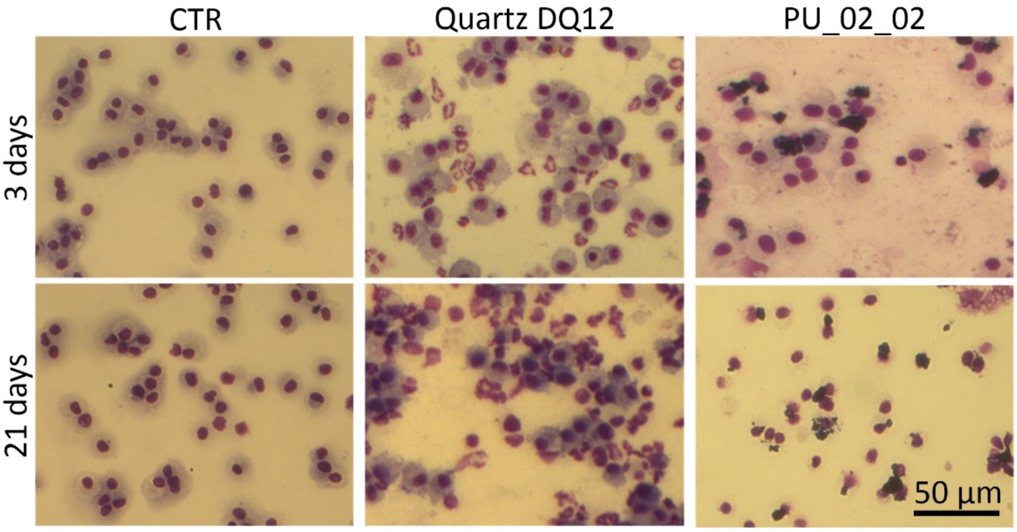

Figure S4 Cytospin preparations of the BALF 3 and 21 d after intratracheal instillation of NaCl (CTR), quartz DQ12 and PU_02_02. CTR: Alveolar macrophages, DQ12: AM intermingled with PMN. PU_02_02: AM contain dark material, interpreted as PU_02_02 particulate matter. Eosinophilic fine granular material is visible on day 3 but no longer on 21.

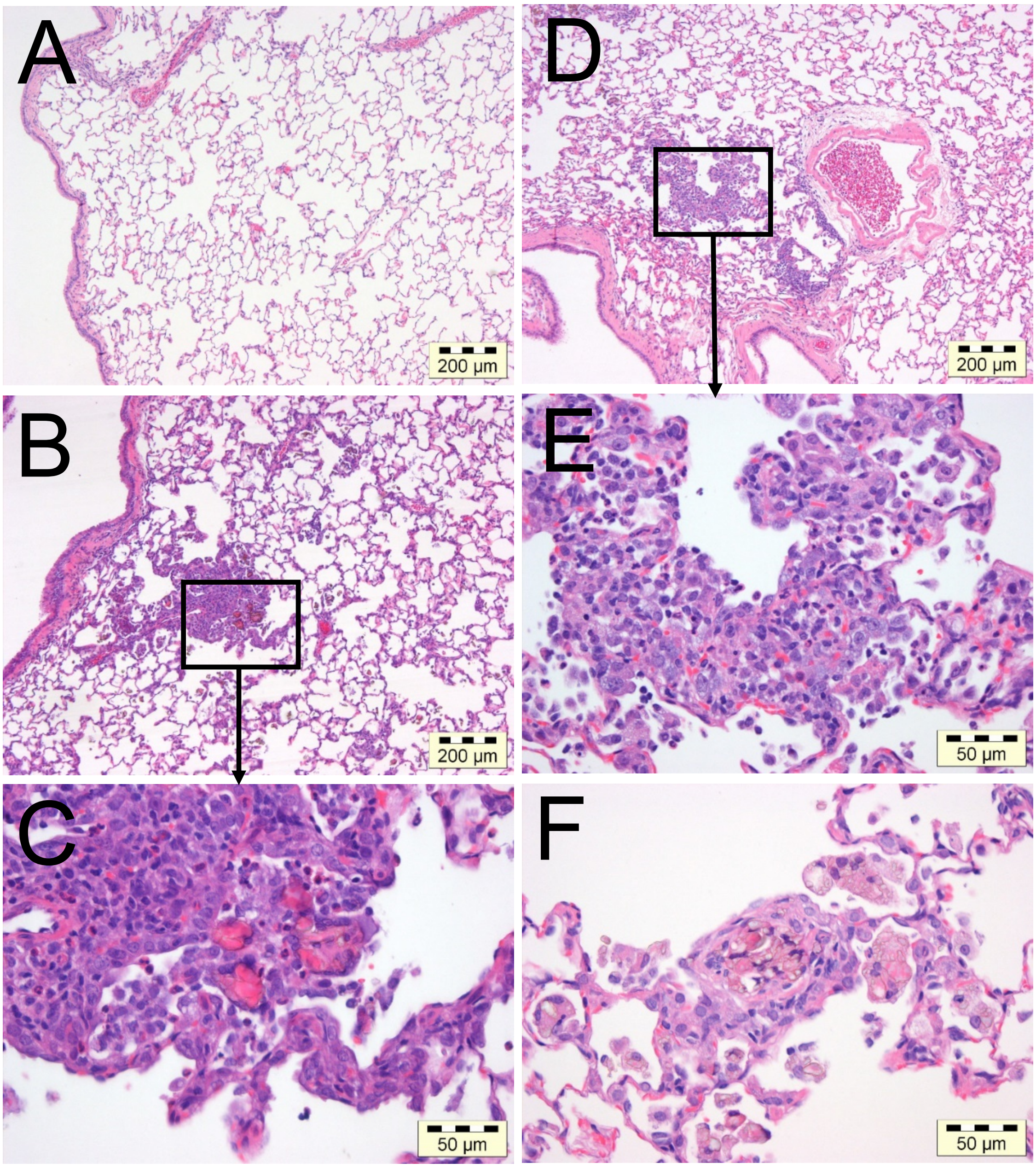

Figure S5 Illustration of histopathological findings in the lung after intratracheal instillation of PU_02_02 (2.4 mg). A: control animal (Hematoxylin Eosin (HE) stain, 40x magnification). B: treated animal 3 days after instillation: minimal to slight granulomatous broncho-interstitial inflammation, minimal to moderate mixed cell infiltration, alveolar foreign material, minimal to moderate alveolar histiocytosis, hypertrophy/hyperplasia of terminal bronchioles, and alveolar lipoproteinosis (HE stain, 40x mag.). C: higher magnification of B (HE stain, 200x mag.). D: treated animal 21 days after instillation: all signs of a slight impairment (granulomatous broncho-interstitial inflammation, alveolar histiocytosis, mixed cell infiltration and hypertrophy/hyperplasia of terminal bronchioles) are still detectable (HE stain, 40x mag.). E: higher magnification of D (HE stain, 200x mag.). E: treated animal 21 days after instillation: foreign material is still present in macrophages (HE stain, 200x mag.).

References in ESI

1. T. Budtova, *Cellulose*, 2019, **26**, 81-121.
2. M. Fricke and M. Elbing, *Journal*, 2014.
3. T. Budtova and P. Navard, *Cellulose*, 2016, **23**, 5-55.
4. A. M. Saeed, P. M. Rewatkar, H. Majedi Far, T. Taghvaee, S. Donthula, C. Mandal, C. Sotiriou-Leventis and N. Leventis, *ACS Applied Materials & Interfaces*, 2017, **9**, 13520-13536.
5. G. Zhang, A. Dass, A.-M. M. Rawashdeh, J. Thomas, J. A. Counsil, C. Sotiriou-Leventis, E. F. Fabrizio, F. Ilhan, P. Vassilaras and D. A. Scheiman, *Journal of Non-Crystalline Solids*, 2004, **350**, 152-164.
6. ISO/TR19057, *ISO/TR*, 2017, **19057**.
7. A. B. Stefaniak, R. A. Guilmette, G. A. Day, M. D. Hoover, P. N. Breysse and R. C. Scripsick, *Toxicology in Vitro*, 2005, **19**, 123-134.
8. M. Marques, R. Loebenberg and M. Almukainzi, *Dissolution Technologies*, 2011, **8**.
